# The Impact of Village Rules and Formal Environmental Regulations on Farmers’ Cleaner Production Behavior: New Evidence from China

**DOI:** 10.3390/ijerph18147311

**Published:** 2021-07-08

**Authors:** Shichun Du, Jing Liu, Zetian Fu

**Affiliations:** 1 School of Public Administration, Shandong Technology and Business University, Yantai 264005, China; 2 School of Business, Ludong University, Yantai 264025, China; jliu@ldu.edu.cn; 3 Yantai Research Institute, China Agricultural University, Yantai 264670, China; fzt@cau.edu.cn

**Keywords:** village rules, formal environmental regulations, agricultural cleaner production, multivariate probit model

## Abstract

Village rules and formal environmental regulations are of great significance for standardizing farmers’ cleaner production behavior, promoting green transformation of agriculture and realizing sustainable development of agriculture. Based on the survey data of 946 farmers in five provinces of China, taking seed coating technology, soil testing and formulated fertilization technology, subsoiling tillage technology, green technology for pest and disease control and straw returning technology as examples, this article empirically analyzes the impact of village rules and formal environmental regulations on farmers’ cleaner production behavior by using the multivariate probit model. When formal environmental regulations are relatively lacking or weak, village rules can be used as a useful supplement to formal environmental regulations to promote farmers’ participation in cleaner production. Based on this, this article argues that the important reason for formal environmental regulations falling into relative system failure is that village rules have not been paid enough attention in promoting farmers’ cleaner production behavior. In the future, we should not only continue to strengthen the role of formal environmental regulations in farmers’ cleaner production, but also cultivate the informal institution represented by the village rules, and build the regulatory system of mutual support between informal institution and formal institution.

## 1. Introduction

China’s agricultural development has made remarkable achievements since the reform and opening up [1]. In 2020, China’s grain output reached 670 million tons, and has increased by about 119.82 percent since 1978 [2]. At the same time, agricultural resources are overexploited due to the long-term high output through high input, and agricultural pollution is aggravated [3]. At present, China has less than 8% of the world’s arable land, but the annual application of chemical fertilizer accounts for more than one third of the world’s total, which is close to twice the internationally recognized upper limit of chemical fertilizer application (225 kg/ha) [4]. The annual application amount of pesticides has exceeded 300,000 tons, and the application intensity has reached 25 kg/ha, which is three times the world average level [4]. Agricultural water consumption accounts for more than 60% of national economic water consumption, and agricultural irrigation water efficiency is only 75% of that of developed countries [5]. The over intensive production mode of farmers not only causes a serious waste of water resources and agricultural chemicals, but also leads to double constraints on use of water and soil resources, which affects the high-quality development ability of agriculture and the income level of farmers. Therefore, it is vital to change the mode of agricultural production and vigorously promote agricultural cleaner production [6].

Agricultural cleaner production is a practical agricultural technology and scientific production management mode, which can not only meet the needs of agricultural production, but also make rational use of resources and protect humans and the environment [7]. Agricultural cleaner production requires the rational use of chemical fertilizers, pesticides, and other inputs, and encourages farmers to carry out soil testing, formulated fertilization technology, and straw returning technology, etc., so as to control agricultural pollution from the source and reduce the risk of agricultural production and service processes to the environment and human beings [8].

Affected by the economic level, urban–rural dual structure, and agricultural development stage, China’s agricultural cleaner production has grown out of nothing and has experienced the process from being simple to becoming comprehensive, from the edge to the mainstream. China’s environmental protection law was first promulgated in 1979. In 1988, the State Environmental Protection Bureau was established, which is the predecessor of the Ministry of Ecological and Environment of the People’s Republic of China. It is responsible for the establishment and improvement of the basic system of ecological environment and the supervision of the prevention and control of environmental pollution. In the past, China’s environmental protection focused on urban and industrial pollution control, and the field of agricultural environment was in a blank or marginal position. Gradually, China attached importance to the development of agriculture and rural areas, and put forward goals for a new and beautiful countryside. However, agricultural environmental governance was still behind in the construction of rural roads, power grids, and water conservancy due to the limitation of agricultural resources. With China entering a new era, the government has deeply realized the importance of agricultural environmental protection, which has created a very powerful condition for agricultural environmental governance [9]. The Chinese government advocates that farmers’ production behavior should be adjusted through the “top-down” governance mode of environmental regulation. However, due to the dispersion, uncertainty, and complexity of China’s rural problems, as well as the heterogeneity of historical and cultural basis and socio-economic development level of each village, the governance effect of government environmental regulation has not been satisfactory and has failed to achieve the expected effect.

Environmental pollution is the “by-product” produced by farmers in agricultural production activities, which revolves around the whole process of agricultural production [10]. According to the Kuznets curve hypothesis of environment, the relationship between environmental quality and agricultural development presents an inverted U-shape, that is, agricultural growth will aggravate environmental pollution in the early stage of agricultural development, but when agricultural growth breaks through the Kuznets turning point, agricultural growth will start to be conducive to environmental protection [11]. Obviously, China has not yet broken through this “inflection point”, and the rapid development of agriculture is still based on the support of environmental resources [3]. Therefore, although formal environmental regulations play an important role in promoting farmers’ cleaner production behavior, the attitude orientation of local government to give priority to agriculture or to protect the environment determines the implementation effect of the formal environmental regulations. Much of the literature also supports this view [12,13]. Due to the promotion tournament governance model in China, local officials pay more attention to crop yield than environmental protection [14]. In addition, the uncertainty and complexity of China’s rural problems and the differences in the historical, cultural, and economic development level of each village also lead to the unsatisfactory effect of government environmental regulations.

New institutional economic theory points out that the binding force of informal institution is often more obvious than that of formal institution because of the contagion continuity of informal institution [15]. Therefore, when formal environmental regulations have failed relatively to provide governance effects on environmental issues, the role of informal institution should not be ignored [16,17]. Informal institution generally consists of religion, culture, customs and interpersonal relationships. Village rules, as an important component of the informal institution, have become an important form of villagers’ autonomy and have been highly praised by many countries [18].

Therefore, the implementation of farmers’ cleaner production is inseparable from the dual forces of village rules and formal environmental regulations. It is necessary to build a complete regulatory system including village rules and formal environmental regulations. To address the research question, a multivariable probit model was constructed to investigate the impact of village rules and formal environmental regulations on farmers’ cleaner production behaviors based on the survey data of 946 farmers in five provinces of China. In the article, there are five kinds of cleaner production technologies to be researched, including seed coating technology, soil testing and formulated fertilization technology, subsoiling tillage technology, green technology for pest and disease control, and straw returning technology. The research results provide a useful reference for the realization of an agricultural cleaner production mode which combines environmental regulations with village rules.

This article makes three key contributions to the field. First, concerning the research objective, farmers would adopt a variety of cleaner production technologies during pre-production, in-production and, post-production because of the complexity of the agricultural production process. However, most of the existing researches focus on one kind of cleaner production behavior, and the research results are not enough to guide the whole process of farmers’ cleaner production practice [19]. The five cleaner production technologies selected in this article can cover the main links of agricultural production, including sowing, fertilization, arable land, management, and harvesting. Second, concerning the research content, most of the existing literature focuses on the impact of formal environmental regulations on agricultural cleaner production [20], and pays less attention to the relationship between village rules and farmers’ cleaner production behavior. In this article, the village rules and formal environmental regulations are brought into a unified research framework for analysis. Third, concerning the research method, the binary probit model is widely used in the previous literature [21]. In reality, farmers have the possibility to choose a variety of cleaner production technologies, and these production technologies are not mutually exclusive, which makes it difficult for the simple binary probit model to accurately reflect the practical problems. In this article, the multivariable probit model is used to analyze farmers’ cleaner production behavior, which improves the accuracy of the estimation results.

This rest of this article is organized as follows. Section 2 conducts a literature review. Section 3 presents the theory and hypotheses. Section 4 describes data sources and methodology. Section 5 analyses the empirical results. Section 6 provides the main findings and policy recommendations.

## 2. Literature Review

### 2.1. Formal Environmental Regulations and Farmers’ Cleaner Production Behavior

The purpose of formal environmental regulations is to protect the environment, encourage and guide farmers to choose a pro-environment agricultural production mode, and punish various behaviors that pollute the environment, so as to coordinate the relationship between environmental protection and economic development [22]. The agricultural ecological environment is a type of social public property. The protection of the agricultural ecological environment has a strong positive externality. However, farmers often need to invest too much capital and technology to adopt cleaner production. Without any economic compensation and incentives, farmers are generally not willing to pay for such public goods, which will lead to the so-called free-rider phenomenon [23]. This requires the government’s formal environmental regulations to promote agricultural cleaner production.

In 1972, the United States first proposed to control non-point source pollution and advocated “Best Management Practices” (BMPs) based on the rationalization of land use. Agricultural cleaner production in Japan, also known as environmental safety agriculture, emphasizes the main role of producers and implements the certification of “Environment-friendly Farmers”. South Korea attaches great importance to the guidance of macro planning, and has put forward the plan of cultivating “Pro-Environment Agriculture”, and established the blueprint and direction of medium and long-term policies. Since the end of the 1980s, the European Union has promoted the “Voluntary Partnership Program” and combining agricultural technology with supporting policies to implement “Good Farming Practices” (GPA), which is linked with direct subsidies. China has formulated and promulgated a series of rules and regulations on agricultural cleaner production since 1992, and the Ministry of Agriculture and Rural affairs of the People’s Republic of China also issued the “Opinions on Accelerating Agricultural Cleaner Production” in 2011. “No. 1 Central Document” has also been stressed many times. However, China’s agricultural cleaner production has not yet been realized on a large scale due to the imperfect policies and regulations and bad management [24].

Many scholars have tried to find a suitable way to control agricultural environmental pollution in China from different angles.

The first perspective is based on Pigouvian tax [25]. Pigouvian tax, named after economist Arthur C. Pigou, is considered to be equal to the value of the negative externality. The main content is that the government levies taxes on polluters who cause environmental pollution, and subsidizes those who reduce or protect the environment, so as to raise funds for environmental pollution control, and force and encourage enterprises to pursue more effective ways to reduce costs and protect the environment [26]. China has officially implemented environmental protection tax since 1 January 2018, which plays an important role in urban environmental pollution control. At present, China’s urban pollution has been curbed, and the urban environment has been greatly improved [27]. However, this kind of measure, mainly based on government taxation, cannot play a role in the treatment of agricultural environmental pollution because agricultural environmental pollution is caused by quantitative change to qualitative change, and the single pollution source does not meet the tax requirements and cannot be taxed [28,29].

The second perspective is based on the “Coase Theorem”. Ronald H. Coase, a British economist, regarded environmental pollution as a kind of property right and proposed that environmental pollution control should be carried out through market mechanisms under the condition of clear property rights [30]. This method is still useful for the pollution control of enterprises and factories, but there would be many problems if we used this for agricultural environmental pollution control [31,32,33]. First of all, how to intervene in the market is a difficult problem to solve at present. Second, how to clarify the property rights. Therefore, it is difficult to solve the problem of agricultural environmental pollution by relying solely on market mechanisms, which need the cooperation of government regulation.

The third perspective is under the theory of “polycentric governance”. Polycentric governance is a model formed by the government, market, and society based on the common goal of environmental governance. It defines rights and responsibilities, and takes control, division of labor, consultation, and other ways to continuously interact to control agricultural environmental pollution [34]. Polycentric governance brings farmers into the main subject of environmental pollution control, but it still does not consider the problem of agricultural environmental pollution control from the perspective of village rules. In addition, there are many obstacles in the mode due to the overlapping rights and unclear responsibilities of governance subjects [35,36]. The main reason is that the focus of current governance methods is on the external sources of agricultural environmental pollution, not from the endogenous nature of agricultural society. This requires the intervention of “rural ecological culture” to solve the endogenous problem of agricultural environmental pollution control.

### 2.2. Village Rules and Farmers’ Cleaner Production Behavior

Due to the obvious externality of environmental governance, one party may benefit while the other party may suffer loss. This loss is not only reflected in the economic level, but also includes emotional, moral, and other factors [37]. Village rules are rooted in farmers’ daily life and interactions, and influence and restrict farmers’ behavior through external pressure. For rural society, village rules often come from the production, life, and communication in the field, which is the internalized code of conduct of villagers. Once someone oversteps, they are scorned, condemned, and punished by the community of the village, making them unable to hold up their heads in the “acquaintance society” of the village [38]. These human relations, rituals, and customs are either explicitly stipulated or established by convention. They may be expressed in words, or handed down by word of mouth. They are either created by human beings or generated naturally. They may be implemented by some specific people or by public opinion and some subtle psychological mechanism [39]. Therefore, in addition to the formal environmental regulations, some scholars have recently tried to explore the impact on environmental governance based on some village rules, such as media reports, traditional culture, public pressure, and ethics [40,41,42]. Gray et al. [43] and Huang et al. [44] pointed out that ethics and external public pressure had a positive impact on farmers’ cleaner production behavior. Clarkson et al. [45] and Xu et al. [46] studied the influence of public opinion supervision on cleaner production behavior, and media reports helped to disclose more detailed environmental information. Village rules help to improve the quality of environmental information disclosure from the perspective of traditional Chinese culture, and form a complementary effect with formal environmental regulations.

## 3. Theory and Hypotheses

### 3.1. Traditional Authority Theory

In an agricultural society, village rules are the main criterion used to restrict people’s behavior [47]. The reason is that village rules are supported by traditional authority. The representatives of traditional authority often come from the elites in the economic and cultural fields. They have a strong binding force on people’s behavior in daily production and life. There are three types of traditional authority in rural areas: First, rural grassroots organizations. Rural grassroots organizations include various organizations at the town and village level, mainly including village Party organizations, village committees, etc. Because of their official recognition and authorization, rural grassroots organizations have authority and force of legitimacy [48]. Second, rural elites. Rural elites are social groups in a dominant position in rural society. With their knowledge, wealth, and identity, they become authorities and play the role of advocating, formulating, and implementing village rules [49]. In the process of rural science and technology demonstration, the relationship between rural elites and farmers shows an “innovation–follow” relationship. Third, the patriarchal clan. Influenced by moral ethics, customs, religious beliefs, and family reverence, the patriarchal clan system often has higher authority among farmers. Village rules can transmit various values through the patriarchal clan system, and then affect the behavior of farmers [39].

Therefore, the following hypothesis is proposed:

**Hypothesis** **1** **(H1).** *Village rules will promote farmers’ cleaner production behavior*.

### 3.2. Externality Theory

Externality is the effect of one economic subject’s behavior on the welfare of another economic subject, which is difficult to be reflected in money or market transactions [26]. An externality can be positive or negative. Agricultural environmental pollution is a well-known negative externality, which can be solved by internalizing the externality. Internalizing the externality includes two modes: the Pigouvian taxes approach and the Coase theorem approach [26,30,50]. Pigouvian tax can overcome negative externalities by the government’s tax collection on the main body of polluting agricultural ecological environment. Subsidies are also one solution to overcoming externalities by encouraging the behavior of protecting the agricultural ecological environment (positive externality). In rural areas, the government tax is mainly on agricultural enterprises, but it cannot play a role for small farmers. The main reason is that the production scale of small farmers does not meet the tax requirements and cannot be taxed. Subsidies are an effective solution [28]. Coase theorem holds that the fundamental reason for externality lies in the unclear definition of property rights and the external problems should be solved by market means, so incentive-based regulation is highly respected [31,50]. However, it is difficult to clarify the property rights because the agricultural production environment is a type of public property. Therefore, the market mechanism has some limitations in solving the problem of agricultural environmental pollution, which needs to be coordinated by government command-and-control regulation and guidance regulation [51,52,53].

Based on the above analysis, this article puts forward hypothesis H2:

**Hypothesis** **2** **(H2).** *Formal environmental regulations will promote farmers’ cleaner production behavior*.

## 4. Methodology and Data Sources

### 4.1. Data Sources

**Figure 1:**
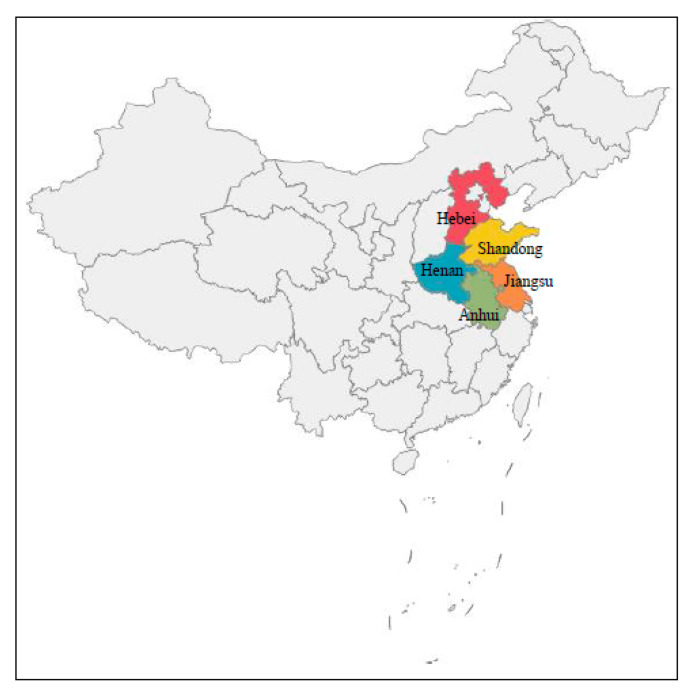
Location map of sample provinces in China.

The data used in this paper were collected from the questionnaire survey of 981 farmers in 92 villages and 45 towns in 15 counties of Shandong, Henan, Hebei, Anhui, and Jiangsu provinces from July to November in 2019. The map shows the location of these provinces as Figure 1. The reason for choosing these five provinces is that they are important agricultural provinces and grain production bases in China. Shandong, Henan, Hebei, Anhui, and Jiangsu provinces are located in the North China Plain of China. The North China Plain is the largest grain producing area in China. The cultivated land rate of Shandong Province is the highest in China. Wheat, corn, and sweet potato are the three staple crops in Shandong Province. Henan Province, with a cultivated area of 6.871 million hectares, is one of the three provinces with grain output exceeding 30 million tons. Hebei Province encircles the capital Beijing. The staple crops in Hebei Province are wheat, corn, rice, and sorghum. The Yangtze River flows through Anhui Province and Jiangsu Province, which can bring abundant water resources and soil suitable for various crops. Jiangsu Province is a famous “land of fish and rice”. It has unique agricultural production conditions and a wide variety of crops.

This survey adopted the method of multi-stage stratified sampling and random sampling. Firstly, three counties were selected in each province, next, three sample towns were selected in each sample county, then, 2–3 villages were randomly selected in each town, and finally, 10 farmers were selected in each village for investigation. In order to ensure the quality of the survey, all the researchers received professional training in the early stage. The questionnaires were asked by the investigators and filled in by themselves. A total of 981 questionnaires were distributed in this survey. After eliminating invalid questionnaires, key information missing questionnaires, logical errors and missing variables, a total of 946 valid questionnaires were collected.

### 4.2. Model

Farmers may adopt a variety of cleaner production technologies at the same time, and there is a certain relationship between these cleaner production technologies. Therefore, a multivariate probit model was used to analyze farmers’ cleaner production behavior [54]. The following Equation (1) represents the model

(1) $$\begin{array}{l} Y_{j}^{*}=\beta_{j}X+\varepsilon_{j}\\ Y_{j}=\{\begin{array}{c} \begin{array}{cc} 0, & if\;Y_{j}^{*}\leq 0 \end{array}\\ \begin{array}{cc} 1, & if\;Y_{j}^{*}>0 \end{array} \end{array} \end{array}$$

where *j* refers to different cleaner production technologies. *X* means the influencing factors of farmers’ cleaner production behavior. $Y_{j}^{*}$ denotes an unobservable latent variable. $Y_{j}\;$ is the final variable result. $Y_{j}=1$ means farmers adopt corresponding cleaner production technology. $\beta_{j}$ is the corresponding estimation coefficient and $\varepsilon_{j}$ is a random disturbance term.

### 4.3. Variable Description

#### 4.3.1. Dependent Variables

According to the definition of the United Nations Environment Programme, five kinds of cleaner production technologies are selected, including seed coating, soil testing and formulated fertilization, subsoiling tillage, green technology for pest and disease control, and straw returning [55].

Seed coating. Seed coating is a type of technology used to promote agricultural production and harvest [56], that is to say, seed coating agents containing insecticides, fertilizers, growth regulators, slow-release agents, and other components are evenly wrapped on the surface of seeds to form a smooth and firm film. With the germination, emergence, and growth of seeds, active ingredients in the seed coating agents are gradually absorbed by plants, which play a role in controlling diseases and pests, promoting growth, and improving crop yield. The coated seeds are buried under the ground after sowing, which can reduce the waste of chemicals and environmental pollution [57].

Soil testing and formulated fertilization. Chemical fertilizer is an important agricultural material that plays an important role in increasing crop yield. However, excessive use of chemical fertilizer has brought serious consequences to China’s environment, agriculture, and economy [58]. Soil testing and formulated fertilization is a low-carbon technology with strong environmental awareness that aims to reduce carbon emissions and improve the ecological environment caused by excessive fertilizer application [59]. Zhen et al. verified the economic feasibility of this technology, and analyzed the beneficial effects from the aspects of production cost, crop yield, fertilizer utilization rate and overall reduction of agricultural pollution [60]. The Chinese government has promoted soil testing and formulated fertilization technology since 2005, but the farmers’ acceptance rate is low [61].

Subsoiling tillage. Subsoil tillage is one of the most effective means to break up a plow pan in agricultural production [62]. As many researchers have reported, subsoil tillage can play important roles in accelerating the infiltration of surface water, reducing ineffective evaporation, and improving the ecological environment for root development and root activities that enhance the anti-stress capacity of plants [63,64]. Varsa et al. found subsoiling tillage could increase crop yield, especially in dry seasons [65].

Green technology for pest and disease control. The use of chemical pesticides has made a great contribution to solving the problem of world food security [66]. However, a large number of inefficient chemical pesticide inputs have caused frequent quality and safety incidents of agricultural products and the deterioration of rural ecological environments, which have seriously affected human health [67,68]. Green technology for pest and disease control has become an effective way to achieve the reduction and substitution of pesticides and the sustainable management of diseases and insect pests because of its excellent technical attributes such as resource saving, being environment-friendly, and human and animal safety [69,70]. Green technology for pest and disease control includes ecological regulation technology, biological control technology, physicochemical deception control technology and scientific medication technology [71,72]. At present, the coverage rate of green technology for pest and disease control of main crops is only 27.2% in China [68]. Only when the net income of adopting green technology for pest and disease control is greater than that of traditional chemical prevention and control, farmers are willing to adopt green technology for pest and disease control [67]. At present, there is no consensus on whether green technology for pest and disease control can improve farmers’ economic benefits [67,73], but it plays an important role in ensuring food security and protecting the ecological environment [72,74].

Straw returning. Crop straw has been the basic energy source of rural life in China for a long time [75]. With the improvement of rural infrastructure and the extensive use of natural gas in recent years, straw as rural energy has been unpopular. Instead, farmers directly burn straw in open fields during the harvest season as a fast and simple treatment method, causing serious seasonal air pollution [76]. Straw returning to field has been widely promoted in China for its advantages of environmental protection and easy implementation. Therefore, when harvesting the main crops, the automatic grinder connected to the harvester can be used to return the straw to the field. The straw can be cut into small pieces and left in the soil. The straw retained in the farmland can increase the storage of organic carbon and nitrogen, so it is possible to improve crop productivity [77,78]. Of course, straw returning has some disadvantages [79], but the rational use of straw returning technology is conducive to improving soil quality and increasing yield, which has been verified in the North China Plain [80].

These cleaner production technologies can cover the main links of agricultural production, such as sowing, fertilization, arable land, management, and harvesting. Whether farmers adopt one of the above-mentioned agricultural cleaner production technologies is selected to measure farmers’ cleaner production behavior.

#### 4.3.2. Independent Variables

Village rules influence farmers’ cleaner production behavior mainly through ideological orientation, disciplinary supervision, transmission, and internalization [42,44,45,46].

Ideological orientation. Ideological orientation refers to the impact of cognition and values on farmers’ production. Village-level organizations set up various model titles to guide farmers’ cleaner production behavior. Rural elites guide farmers’ cleaner production behavior by taking the lead in demonstration.

Disciplinary supervision. Disciplinary supervision refers to the use of economic punishment, public opinion control, and other measures to achieve the purpose of restraining farmers’ production behavior by means of village-level organizations.

Transmission and internalization. Transmission and internalization refers to the process of realizing the transmission and diffusion of values by relying on authoritative subjects such as the patriarchal clan system and rural elites. It is mainly realized through the interaction between farmers.

Corresponding to the externality theory, this article also sets the following three indicators for formal environmental regulations [19,28,33,53].

Command-and-control regulation. This means the government makes laws, regulations, and policies to restrict farmers’ production behavior. Once farmers deviate from the regulation goal, they face accountability and punishment. After farmers weigh up the cost of violation, economic rationality will urge them to comply with the regulation goal and gradually change to the direction of cleaner production.

Incentive-based regulation. This means the government reduces the transaction cost of farmers’ participation in cleaner production by issuing economic subsidies and tax relief so as to mobilize the enthusiasm of farmers in cleaner production.

Publicity-and-guidance regulation. This means the government carries out publicity and education on environmental protection, so as to guide farmers on how to regulate their production behavior.

#### 4.3.3. Control Variables

In this article, we set five control variables. They are farmers’ personal characteristics, family characteristics, social characteristics, market level, and regional level. Farmers’ personal characteristics mainly include age and education level [81,82]. Farmers with different age and education levels have different cognitions of cleaner production due to different labor input in agricultural production. Family characteristics mainly include the number in the family agricultural labor force, family income, and farm size [19]. Agricultural production decision making is a kind of family behavior. Family characteristics reflect the human, land, capital, and other factors endowment of family agricultural production, which determines whether farmers are willing to adopt new technologies for cleaner production. Social characteristics include whether the family has civil servants and whether they participate in agricultural cooperatives [83,84]. Farmers participating in agricultural cooperatives may carry out agricultural cleaner production under the leadership of the organization. At the market level, the benefit of cleaner production is included in the model [85]. In addition, considering the heterogeneity of each region, this article also sets up four regional dummy variables. The definition and assignment of all variables are shown in Table 1.

The indicators of village rules and formal environmental regulations in Table 1 are assigned based on a five-point Likert scale from very important to very neglected or from very large to very small. Considering farmers may strategically “understate” or politely “overstate” their real thoughts [86,87], this article re-evaluates the variables. That is to say, “very neglected”, “neglected”, and “general” are merged into “Neglected” and assigned to 0, and “very important” and “important” are merged into “Important” and assigned to 1; “very small”, “small”, and “general” are merged into “Small” and assigned to 0, and “very strong” and “strong” are merged into “Strong” and assigned to 1.

## 5. Results and Discussion

We used Stata software to build a multivariable probit model in the following ways: Firstly, considering the impact of village rules on farmers’ cleaner production behavior, we obtained regression model 1. The regression results are shown in Table A1. Secondly, considering the impact of formal environmental regulations on farmers’ cleaner production behavior, we obtained regression model 2. The regression results are shown in Table A2. Finally, considering the impact of rural regulations and formal environmental regulations on farmers’ cleaner production behavior, model 3 was obtained. Since model 3 includes all the key indicators considered in this article, the following mainly analyzes the estimation results of model 3 (see Table 2). Overall, the results show that the fitting results are good because the value of Wald chi^2^ is very significant at 1% level.

### 5.1. The Impact of Village Rules

From model 3 (Table 2), ideological orientation, disciplinary supervision, and transmission and internalization passed the significant test, which shows that village rules can effectively encourage the occurrence of farmers’ cleaner production behavior.

Ideological orientation has a significant positive impact on seed coating technology (*β* = 0.467, *p* < 0.01), green technology for pest and disease control (*β* = 0.462, *p* < 0.01), and straw returning technology (*β* = 0.627, *p* < 0.01). Coated seeds are usually provided by several service organizations, such as trusteeship organizations, general agricultural materials dealers, etc., which are easy to buy, and this technology can prevent and control diseases and pests at seedling stage and improve crop yield. Straw returning technology can not only alleviate the soil structure damage and rural air pollution caused by straw burning but also enhance the disease resistance of crops in the next season and save fertilizer. Green technology for pest and disease control helps farmers reduce pesticide use by 30–50%, ensure the quality and safety of agricultural products, meet the standards of pollution-free and green food, and bring value-added effect to farmers [85]. Therefore, these three technologies fit the farmers’ own interests and are easy to be adopted and implemented by farmers. Through the establishment of the cleaner production honorary title, the ideological orientation not only provides farmers with examples but also meets their honor demands, thus greatly improving the probability of farmers adopting seed coating, straw returning, and green technology for pest and disease control. Ideological orientation does not significantly encourage farmers to adopt soil testing and formulated fertilization technology and subsoiling tillage technology. The possible reasons are as follows: most of the soil testing and formulated fertilization technology are provided by the land trusteeship organization, the testing methods are lagging behind, the analysis speed is slow, and the testing accuracy is often poor, so the farmers’ trust in the technology is not high and the farmers’ willingness to adopt it is low. Subsoiling tillage not only requires high tractor power but also the price of high-quality subsoiler is not low, resulting in the high price of the whole set of subsoiling equipment. The fuel consumption of subsoiling tillage plus the whole set of equipment will result in a higher operation cost fed back to farmers. For the consideration of cost minimization and agricultural risk, farmers will more rationally choose whether to adopt the two techniques. Although ideological orientation can perform a demonstration and honor-stimulating effect on farmers, it cannot offset farmers’ worries about the increase of cost and risk at this time. Therefore, ideological orientation has no obvious effect on promoting farmers to adopt soil testing and formulated fertilization technology and subsoiling tillage technology.

Disciplinary supervision has a significant positive impact on farmers’ adoption of soil testing and formulated fertilization technology (*β* = 0.378, *p* < 0.01) and straw returning technology (*β* = 0.453, *p* < 0.05). Village rules not only make corresponding norms for farmers’ cleaner production behavior but also formulate a series of punishment measures linked with farmers’ potential interests. If farmers have some non-clean production behavior such as straw burning and indiscriminate application of chemical fertilizer, it will pollute the soil, water, and air of the village. With the popularization of environmental protection policies, villagers also pay attention to their own living environment. Villagers may criticize such behavior or even impose economic punishment. In this case, in order to avoid possible punishment, farmers may tend to adopt soil testing and formulated fertilization technology and straw returning technology. Disciplinary supervision cannot significantly encourage farmers to adopt subsoiling tillage technology and green technology for pest and disease control. The possible reason is that it will not significantly affect the ecological environment of the whole village if farmers do not adopt this technology. The reason why disciplinary supervision fails to encourage the farmers to adopt the coated seeds technology is that farmers find that the crops using the seed coating technology has strong insect resistance and their yield increases significantly. Through word of mouth, farmers share knowledge and voluntarily adopt the clean technology. Therefore, the role of disciplinary supervision is not obvious.

Transmission and internalization had significant positive effects on seed coating (*β* = 0.492, *p*< 0.01), soil testing and formulated fertilization (*β* = 0.356, *p* < 0.05), and subsoiling tillage (*β* = 0.556, *p* < 0.01). In rural areas, farmers communicate frequently and know each other’s specific behaviors in the process of agricultural production. Driven by herd mentality, farmers are vulnerable to the influence of other farmers around, resulting in herding behavior. Seed coating, soil testing and formulated fertilization, and subsoiling tillage are conducive to improving crop emergence rate and yield, all of which have the characteristics of a quick effect and short action period. Therefore, once some farmers recognize and accept them, the transmission and internalization of village rules may promote other farmers to adopt those technologies through the interaction among farmers. Transmission and internalization cannot significantly promote farmers to adopt straw returning technology and green technology for pest and disease control. There is a premise that transmission and internalization can encourage farmers to adopt a clean technology, that is, if the technology has a faster or better effect. Straw returning technology is a type of intertemporal agricultural production technology that has a long effective time, slow-release and uncertain output, and does not fit the psychology and demand of farmers’ current input and current income. Green technology for pest and disease control, such as biological control, is not as quick as chemical control. Artificial propagation of beneficial organisms is more difficult, few species can be used to release a large number of natural enemy insects, and most of the natural enemy insects have a narrow range of action [67,69]. Therefore, transmission and internalization has no obvious effect on encouraging farmers to adopt straw returning technology and green technology for pest and disease control.

Therefore, it can be concluded that village rules can replace part of the role of formal environmental regulations in promoting farmers’ cleaner production behavior. To fully demonstrate this inference, this article will further analyze the interaction between village rules and formal environmental regulations.

### 5.2. The Impact of Formal Environmental Regulations

From model 3 (see Table 2), it can be seen that the command-and-control regulation does not pass the significance test, which indicates that the current formal environmental regulations are not effective in restraining farmers’ behavior, while the incentive-based regulation and publicity-and-guidance regulation pass the significance test, which have a positive impact on farmers’ cleaner production behavior.

Command-and-control regulation in model 3 does not pass the significance test, which shows that the government’s supervision and management of agricultural cleaner production is in a situation of long-term failure, even if the government has stipulated the punishment measures on pollution the actual implementation effect is very poor. The failure of the government management system will gradually become a mere formality in the long-term repeated game [44], and it will be more difficult to effectively regulate the farmers’ pollution behavior. Thus, the command-and-control regulation will find it difficult to have a good effect on farmers’ cleaner production behavior.

Incentive-based regulation has significant positive effects on seed coating (*β* = 0.503, *p* < 0.01), soil testing and formulated fertilization (*β* = 0.519, *p* < 0.01), subsoiling tillage (*β* = 0.629, *p* < 0.05), and green technology for pest and disease control (*β* = 0.643, *p* < 0.01). These technologies belong to the type that increase capital and labor. If farmers adopt these technologies, they will face a certain cost investment. However, the government’s subsidies to farmers not only offset the increased costs but also bring additional transfer income to farmers, expand their interest space, and effectively mobilize the enthusiasm and initiative of farmers in green production. However, incentive-based regulation cannot significantly prompt farmers to adopt straw returning technology. On the one hand, China’s decentralized small-scale land management mode makes the fields of each household separate from each other. Fragmented plots are not conducive to the operation of machinery such as crop harvesters and the harvesting cost is high. On the other hand, the capital investment of purchasing the harvester, baler, and other equipment needed for straw returning is large. In the survey, some farmers reported that the current subsidy for straw returning is only more than 10 yuan per mu [21]. Compared with the equipment and electricity cost for straw returning, this is too small to compensate farmers for the cost of adopting the technology. Therefore, the effect of incentive-based regulation on promoting farmers to adopt straw returning technology is not obvious.

Publicity-and-guidance regulation has a significant positive impact on the five cleaner production behaviors. Because those five kinds of cleaner production technology are conducive to the protection and governance of rural ecological environment, timely publicity and education on environmental protection by the government can encourage farmers to realize the environmental benefits of their production behaviors and deepen their cognition and understanding of cleaner production technology, so as to strengthen farmers’ concept of clean production and increase the possibility of farmers’ cleaner production behaviors.

Therefore, it can be concluded that command-and-control regulation has not passed the significance test, which indicates that formal environmental regulations are not satisfactory in constraining farmers’ cleaner production behavior, and there is a phenomenon of relative institutional failure. North pointed out that the role of formal environmental regulations cannot be successful without the informal institution, and the formal institution must be supplemented and developed by the informal institution [38]. From this point of view, the reason why China’s current environmental regulations fall into “relative institution failure” is that they ignore or belittle the role of informal institutions. Village rules have already formed an internal spontaneous order in rural areas, and gradually penetrated into all aspects of farmers’ life and production. The spontaneous order formed under the “acquaintance society” has increased the governance difficulty of formal environmental regulations and impacted on the effect of formal environmental regulations.

### 5.3. The Impact of Control Variables

In terms of the individual characteristics of farmers, age has a significant negative impact on soil testing and formulated fertilization (*β* = −0.015, *p* < 0.05), subsoiling tillage (*β* = -0.011, *p* < 0.05), and green technology for pest and disease control (*β* = −0.012, *p* < 0.1), as shown in Table 2. The older the farmers are, the lower their ability to respond to, understand, and accept new things, and their learning motivation and passion are not as good as young farmers [44]. The education level of farmers has a significant positive impact on the adoption of soil testing and formulated fertilization (*β* = 0.331, *p* < 0.01). The education level of farmers determines the collection and processing of advanced agricultural clean technology information and their awareness of potential risks. Farmers with a higher education level are more willing to adopt new technology and participate in the innovation process of new technology, so as to promote technological innovation and technology diffusion [22,39]. However, in reality, especially for household farmers, due to the limitation of their own educational level, they often ignore the environmental protection and do not master the new agricultural clean technology [29]. Therefore, the government needs to vigorously develop rural education, especially strengthening the propaganda and technical guidance of agricultural cleaner production knowledge for farmers, and improve the ability of agriculture to use advanced clean technology.

In terms of household characteristics, the larger the family cultivated farm size, the easier it is to adopt subsoiling tillage technology (*β* = 0.479, *p* < 0.01) and straw returning technology (*β* = 0.456, *p* < 0.01), which is related to the need for continuous mechanized operation of these two technologies [29]. It is often easier for large grain growers to adopt these two technologies.

In terms of social characteristics, farmers participating in agricultural cooperatives are more likely to adopt soil testing and formulated fertilization technology (*β* = 0.569, *p* < 0.05). Therefore, the government needs to continue to promote the organizational advantages of cooperatives, carry out targeted publicity, and improve the operability in the promotion of soil testing and formulated fertilization technology.

### 5.4. Interaction between Village Rules and Formal Environmental Regulations

There are some effects on farmers’ cleaner production behavior of the interaction between village rules and formal environmental regulations (see Table 3). Only part of the regression results are shown in Table 3. This article gives equal weight to the specific indicators, and we find the cross coefficient (*β* = −1.232) between the two for straw returning has a significant negative effect on farmers’ cleaner production behavior, which indicates there is a substitution relationship between village rules and formal environmental regulations. That is, in the case of the weak role of formal environmental regulations, village rules may replace these regulations to promote farmers’ participation in cleaner production. Because the control cost of the government is high, village rules can not only save transaction costs but also be closer to the needs of farmers. Therefore, in the case of the poor effect of formal environmental regulations, village rules can play the role of replacing them.

### 5.5. Robustness Test

Considering the elderly do not have the ability to engage in agricultural production, this article removed the samples of the elderly over 60 years old, and carried out the multivariate probit regression again. The results were consistent with the regression results of all samples, which shows that the model estimation results have good robustness.

## 6. Conclusions and Policy Implications

Based on the survey data of 946 farmers in five provinces of China, taking seed coating technology, soil testing and formulated fertilization technology, subsoiling tillage technology, green technology for pest and disease control, and straw returning technology as examples, this article empirically analyzes the impact of village rules and formal environmental regulations on farmers’ cleaner production behavior by using the multivariate probit model. The main findings are as follows: Both village rules and formal environmental regulations can promote farmers’ cleaner production behavior. Among them, ideological guidance, disciplinary supervision and transmission and internalization in village rules can promote the occurrence of farmers’ cleaner production behavior, and incentive-based regulation and publicity-and-guidance regulation in formal environmental regulations also play a positive role in farmers’ cleaner production behavior. The command-and-control regulation in formal environmental regulations fails to pass the significance test, which indicates that there is a phenomenon of “relative institutional failure” in the current formal environmental regulations, and the effect is not good in constraining farmers’ production behavior. Village rules and formal environmental regulations have some interactive effects on farmers’ green production behavior. When formal environmental regulations are relatively lacking or weak, village rules can be used as a useful supplement to formal environmental regulations to promote farmers’ participation in cleaner production.

Based on these findings, several policy implications are proposed. In addition to continuing to strengthen the restriction of formal environmental regulations on farmers’ behavior, we also need to pay more attention to the impact of village rules on farmers’ behavior, coordinate the relationship between village rules and formal environmental regulations in rural environmental governance and farmers’ behavior norms, and finally build a mutual support system between village rules and formal environmental regulations. First, strengthen the role of formal environmental regulations in farmers’ cleaner production. To formulate and perfect formal environmental regulations in line with the basic situation of rural areas in China, integrate them into rural society according to local conditions, and meet the needs of rural development and agricultural production in China. At the same time, we should improve the publicity and popularization of formal environmental regulations, and enhance farmers’ awareness and understanding of environmental regulations, so as to give full play to the role of environmental regulations. Second, we should actively cultivate village rules and give full play to their role in promoting cleaner production of farmers. We should appropriately select, integrate, and perfect the contents of village rules, extract the essence and remove the dross, so as to enhance the feasibility of village rules. Within the framework and constraints of formal environmental regulations, we should give full play to the role of village rules, and cultivate a good cultural soil and support for formal environmental regulations.

## Figures and Tables

**Table 1:** Descriptive statistics of variables.

Variable	Description	Definition	Mean	Std. Dev.
Cleaner production behavior of farmers	Do you adopt seed coating technology?	1 = Yes, 0 = No	0.29	0.45
	Do you adopt soil testing and formulated fertilization	1 = Yes, 0 = No	0.19	0.41
	Do you adopt subsoiling tillage?	1 = Yes, 0 = No	0.25	0.45
	Do you adopt green technology for pest and disease control?	1 = Yes, 0 = No	0.27	0.44
	Do you adopt straw returning?	1 = Yes, 0 = No	0.89	0.29
Ideological orientation	Do you attach importance to the honorary title of cleaner production in the village?	1 = Important, 0 = Neglect	0.35	0.45
Disciplinary supervision	The impact of punishment for non-cleaner production in the village on you	1 = Strong, 0 = Small	0.61	0.47
Transmission and internalization	The impact of cleaner production behavior of others in the village on you	1 = Strong, 0 = Small	0.52	0.51
Command-and-control	The control degree of the government’s environmental regulations on you	1 = Strong, 0 = Small	0.68	0.46
Incentive-based	The impact of environmental subsidies provided by the government on you	1 = Strong, 0 = Small	0.76	0.45
Publicity-and-guidance	The impact of the government’s environmental protection propaganda on you	1 = Strong, 0 = Small	0.84	0.40
Control variables	Age	Years	57.71	5.33
	Education level	Years	6.17	1.23
	The number of family agricultural labor force	Persons	2.32	0.97
	Family income	Ten thousand Yuan	8.19	4.17
	Farm size	Mu	6.92	12.72
	Are there civil servants in the family?	1 = Yes, 0 = No	0.14	0.32
	Are you a member of an agricultural cooperative?	1 = Yes, 0 = No	0.24	0.37
	Benefit of cleaner production	1 = Yes, 0 = No	0.37	0.41
	Regional dummy variables	1 = Henan, 0 = Else	0.18	0.37
		1 = Shandong, 0 = Else	0.21	0.39
		1 = Jiangsu, 0 = Else	0.20	0.40
		1 = Anhui, 0 = Else	0.22	0.39

**Table 2:** Regression results of multivariate probit model (model 3).

Variable		Coefficient				
		Seed Coating	Soil Testing and Formulated Fertilization	Subsoiling Tillage	Green Technology for Pest and Disease Control	Straw Returning
Village rules	Ideological orientation	0.467 *** (0.122)	0.044 (0.113)	0.025 (0.126)	0.462 *** (0.123)	0.627 *** (0.121)
	Disciplinary supervision	0.052 (0.018)	0.378 *** (0.094)	0.061 (0.123)	0.057 (0.112)	0.453 ** (0.113)
	Transmission and internalization	0.492 *** (0.143)	0.356 ** (0.117)	0.556 *** (0.132)	0.183 (0.101)	0.263 (0.118)
Environ. regulations	Command-and-control	0.051 (0.101)	–0.141 (0.161)	0.029 (0.117)	–0.193 (0.122)	–0.191 (0.132)
	Incentive-based	0.503 *** (0.171)	0.519 *** (0.176)	0.629 ** (0.111)	0.643 *** (0.122)	0.242 (0.129)
	Publicity-and-guidance	0.475 *** (0.141)	0.347 *** (0.111)	0.391 ** (0.121)	0.412 *** (0.147)	0.401 *** (0.117)
Control variables	Age	0.004 (0.005)	–0.015 ** (0.005)	–0.011 ** (0.005)	–0.012 * (0.004)	–0.024 (0.102)
	Education level	0.049 (0.016)	0.331 *** (0.011)	0.111 (0.017)	0.121 (0.012)	0.091 (0.014)
	Labor force	0.017 (0.003)	0.011 (0.004)	0.019 (0.002)	0.021 (0.005)	–0.011 (0.003)
	Family income	0.011 (0.002)	0.001 (0.002)	–0.012 (0.004)	0.009 (0.001)	0.002 (0.001)
	Farm size	0.008 (0.041)	0.058 (0.046)	0.479 *** (0.045)	0.069 (0.029)	0.456*** (0.026)
	Civil servants	0.029 (0.098)	0.099 (0.123)	0.084 (0.112)	0.089 (0.097)	0.032 (0.102)
	Agricultural cooperative	0.099 (0.231)	0.569 ** (0.232)	0.141 (0.109)	0.149 (0.201)	0.017 (0.221)
	Benefit of cleaner production	0.169 (0.092)	0.042 (0.123)	0.092 (0.172)	0.147 (0.118)	0.197 (0.147)
	Regional dummy variables	Controlled				
Prob > chi^2^		0.000				
Wald chi^2^		536.67				

Note: standard errors in parentheses, *** *p* < 0.01, ** *p* < 0.05, * *p* < 0.1.

**Table 3:** Interaction between village rules and formal environmental regulations.

Variable	Seed Coating	Soil Testing and Formulated Fertilization	Subsoiling Tillage	Green Technology for Pest and Disease Control	Straw Returning
Village rules	–0.432 (0.541)	0.564 * (0.342)	–0.657 (0.321)	0.543 (0.231)	1.213 ** (0.452)
Environ. regulations	–0.087 (0.076)	0.654 * (0.453)	–0.586 (0.354)	0.612 (0.421)	0.632 ** (0.213)
Village rules × Environ. regulations	0.765 (0.342)	–0.324 (0.187)	0.876 (0.587)	–0.432 (0.319)	–1.232 ** (0.583)

Note: standard errors in parentheses, ** *p* < 0.05, * *p* < 0.1.

## Data Availability

The data presented in this study are available on request from the corresponding author.

## Appendix A

**Table A1:** Regression results of multivariate probit model (model 1).

Variable		Coefficient				
		Seed Coating	Soil Testing and Formulated Fertilization	Subsoiling Tillage	Green Technology for Pest and Disease Control	Straw Returning
Village rules	Ideological orientation	0.449 *** (0.101)	0.035 (0.104)	0.037 (0.112)	0.456 *** (0.102)	0.619 *** (0.119)
	Disciplinary supervision	0.041 (0.014)	0.362 *** (0.191)	0.112 (0.009)	0.059 (0.098)	0.448 *** (0.111)
	Transmission and internalization	0.502 *** (0.134)	0.397 *** (0.109)	0.609 *** (0.123)	0.192 (0.092)	0.274 (0.101)
Control variables	Age	0.003 (0.004)	–0.014 (0.005)	–0.011 (0.004)	–0.011 (0.004)	–0.024 (0.101)
	Education level	0.048 (0.016)	0.331 *** (0.011)	0.112 (0.018)	0.122 (0.013)	0.092 (0.013)
	Labor force	0.018 (0.002)	0.012 (0.003)	0.019 (0.002)	0.021 (0.005)	–0.011 (0.003)
	Family income	0.023 (0.002)	0.011 (0.002)	–0.011 (0.004)	0.008 (0.001)	0.003 (0.001)
	Farm size	0.101 (0.041)	0.002 (0.002)	0.478 *** (0.044)	0.068 (0.028)	0.455 *** (0.023)
	Civil servants	0.031 (0.089)	0.098 (0.122)	0.094 (0.112)	0.124 (0.096)	0.042 (0.111)
	Agricultural cooperative	0.102 (0.209)	0.572 *** (0.233)	0.145 (0.108)	0.148 (0.202)	0.028 (0.229)
	Benefit of cleaner production	0.156 (0.101)	0.059 (0.124)	0.093 (0.171)	0.245 (0.119)	0.207 (0.151)
	Regional dummy variables	Controlled				
Prob > chi^2^		0.000				
Wald chi^2^		471.32				

Note: standard errors in parentheses, *** *p* < 0.01.

**Table A2:** Regression results of multivariate probit model (model 2).

Variable		Coefficient				
		Seed Coating	Soil Testing and Formulated Fertilization	Subsoiling Tillage	Green Technology for Pest and Disease Control	Straw Returning
Environ. regulations	Command-and-control	0.096 (0.093)	–0.112 (0.152)	0.039 (0.102)	–0.182 (0.102)	–0.169 (0.111)
	Incentive-based	0.578 *** (0.167)	0.621 *** (0.161)	0.721 *** (0.103)	0.742 *** (0.103)	0.343 (0.122)
	Publicity-and-guidance	0.596 *** (0.122)	0.459 *** (0.101)	0.471 ** (0.104)	0.541 *** (0.139)	0.501 *** (0.102)
Control variables	Age	0.004 (0.005)	–0.015 (0.005)	–0.011 (0.004)	–0.011 (0.003)	–0.025 (0.003)
	Education level	0.049 (0.016)	0.332 *** (0.012)	0.111 (0.018)	0.122 (0.011)	0.092 (0.013)
	Labor force	0.017 (0.003)	0.012 (0.004)	0.021 (0.003)	0.022 (0.004)	–0.011 (0.003)
	Family income	0.011 (0.002)	0.002 (0.002)	–0.013 (0.004)	0.009 (0.001)	0.005 (0.001)
	Farm size	0.007 (0.039)	0.061 (0.045)	0.482 *** (0.045)	0.071 (0.031)	0.458 *** (0.027)
	Civil servants	0.035 (0.101)	0.132 (0.129)	0.121 (0.112)	0.109 (0.099)	0.097 (0.129)
	Agricultural cooperative	0.079 (0.192)	0.583 *** (0.312)	0.168 (0.111)	0.157 (0.202)	0.016 (0.201)
	Benefit of cleaner production	0.171 (0.102)	0.039 (0.103)	0.111 (0.173)	0.152 (0.108)	0.199 (0.149)
	Regional dummy variables	Controlled				
Prob> chi^2^		0.000				
Wald chi^2^		483.23				

Note: standard errors in parentheses, *** *p* < 0.01, ** *p* < 0.05.
